# Community Pharmacists’ Knowledge Regarding Donepezil Averse Effects and Self-Care Recommendations for Insomnia for Persons with AD

**DOI:** 10.3390/pharmacy5030042

**Published:** 2017-07-28

**Authors:** Marketa Marvanova, Paul Jacob Henkel

**Affiliations:** 1 Department of Pharmacy Practice, School of Pharmacy/College of Health Professions, North Dakota State University, Department 2650, P.O. Box 6050, Fargo, ND 58108-6050, USA; 2 Department of Geographical and Historical Studies, University of Eastern Finland, FI-80101 Joensuu, Finland; suomenpoika@yahoo.com

**Keywords:** community pharmacist, pharmaceutical care, Alzheimer’s disease, cognitive enhancer, acetylcholinesterase inhibitor, donepezil, rivastigmine, anticholinergic agent

## Abstract

Alzheimer’s disease (AD) impacts millions of individuals worldwide. Since no cure is currently available, acetylcholinesterase inhibitors are symptomatic therapy. This study assessed community pharmacists’ knowledge regarding donepezil adverse effects (AEs) and self-care recommendations for insomnia management for persons with AD treated with rivastigmine. This is a cross-sectional, standardized telephone survey of community pharmacists (*n* = 862) in three study areas: West Virginia, North Dakota/South Dakota, and Southern Oregon/Northern California. Pharmacists’ degree, sex, and pharmacists’ AD-related knowledge were assessed. In-stock availability of donepezil and rivastigmine formulations was assessed. Analyses were performed using Stata 10.1. Only 31.4% pharmacists were able to name ≥2 donepezil AEs. Only four donepezil AEs were named by at least 13% of pharmacists: nausea (36.1%), dizziness (25.1%), diarrhea (15.0%), and vomiting (13.9%). All other AEs were named by fewer than 7% of respondents. Only 62.9% of pharmacists (*n* = 542) provided appropriate recommendations: melatonin (40.3%), referral to physician (22.0%), or sleep hygiene (0.6%). Over 12% of pharmacists (*n* = 107) provided inappropriate recommendations (anticholinergic agent or valerian root) and 21.5% of pharmacists were unable to provide any recommendation. We identified significant gaps in community pharmacists’ knowledge regarding donepezil AEs and non-prescription insomnia recommendation needing significant improvement to ensure high-quality AD-related care.

## 1. Introduction

Alzheimer’s disease (AD) is the most common type of irreversible dementia causing 70% of dementia cases among persons over the age of 70 [1,2]. About 50 million people worldwide are diagnosed with AD, 4.5 million residing in the United States (US); and this number is expected to rise in the coming decades [1,2,3]. Advanced age is the greatest risk factor for development of late-onset AD [2]. In the US, this will mean an estimated 7.1 million persons with AD in 2025 [1]. Currently, no disease-modifying therapy is available, so treatment is symptomatic utilizing cognitive enhancers from two pharmacologic classes: acetylcholinesterase inhibitors (AChEIs) (i.e., donepezil, galantamine, rivastigmine), and NMDA-receptor antagonist (memantine) [4,5,6,7,8,9]. Use of AChEIs in persons with AD can have modest beneficial effects on cognitive, global, daily functioning [10]. However, caring for a person with AD requires more than medications to achieve maximum therapeutic benefits.

Pharmacists are medication experts, responsible for ensuring safe and effective medication use in seeking positive treatment outcomes [11,12,13]. Pharmacists practicing in local community pharmacies are among the most accessible healthcare professionals and can play a significant role in provision of primary care for community-dwelling individuals. One-third of individuals with dementia are estimated to be living at home [1]; therefore, they, their family members, or caregivers are likely to be regular clients of local community pharmacies. In the US, states such as West Virginia (WV), North Dakota (ND), and South Dakota (SD), as well as large areas of California and Oregon, are predominantly rural with elderly populations above the national average. Given pharmacists’ medication expertise and role in chronic disease management, and the growing population of older adults in the US [14], pharmacists should possess the requisite knowledge to provide high-quality, contemporary care for AD. 

All community pharmacists should be able to counsel patients on common disease states and disease management, treatment expectations, drug adverse effects (AEs), and interactions. They also should provide safe and effective self-care recommendations alongside general medication dispensing. Currently, no matter where community pharmacists practice, they are likely to serve persons with AD and/or their caregivers. Therefore, it is necessary for pharmacists to have sufficient knowledge and appropriate skills for providing AD-related care [15,16,17]. Recent evidence suggests improvement in pharmacists’ knowledge regarding AD and its pharmacological management is needed [18,19,20].

The study objectives were to understand the knowledge of community pharmacists regarding donepezil AEs and self-care recommendations for insomnia for persons with AD treated with rivastigmine. This knowledge is intended to serve as an indication of potential quality of actual AD-related services in the community that a community-dwelling person with AD, a family member, or a caregiver would receive.

## 2. Materials and Methods

### Study Design

This is a cross-sectional study of community pharmacists and pharmacies utilizing a standardized telephone survey, revised from Marvanova and Henkel [18]. The study population consists of a 100% sample of pharmacies in three high-elderly study areas: WV (*n* = 502), ND (*n* = 179), and SD (*n* = 180), and select counties of Northern California (N.CA.) (*n* = 212), and Southern Oregon (S.OR.) (*n* = 93). A list of community pharmacies in surveyed areas was obtained from individual states’ Boards of Pharmacy. One pharmacist was interviewed from each pharmacy. The research study was approved as exempt by the University Institutional Review Board. 

Pharmacies were cold contacted by telephone during business hours between August and October 2014. Prospective participants were informed that information provided would be used for academic research purposes only, and that participation was voluntary and would remain anonymous. If the pharmacist was unavailable, contact was re-attempted on the same day or a later date. If the time was inconvenient, the pharmacist was provided the opportunity to select a time/day when they would be available. Pharmacists were not informed about the survey questions prior to actual administration as we wanted to assess knowledge without allowing for preparation. Knowledge data was gathered by speaking directly to a pharmacist, taking less than two minutes, but without restriction on interview length. In-stock availability of cognitive enhancers was then obtained from the pharmacist, but if the pharmacist was busy, pharmacy technicians were allowed to provide this information. Two trained, fourth-year pharmacy student assistants obtained pharmacist and pharmacy demographic information and then asked questions on pharmacists’ knowledge regarding AChEI AE(s) and self-care recommendation for insomnia in individual with AD treated with an AChEI. These items were assessed using open-ended questions: “What are the most important AEs to counsel a new patient on regarding donepezil?” and “What non-prescription (non-Rx) recommendation would you provide for a dementia patient currently using rivastigmine patch, for his/her insomnia?” respectively. We assessed in-stock availability of several donepezil formulations (10 mg tablet, 10 mg orally-disintegrating tablet, and donepezil 23 mg tablet), and rivastigmine formulations (9.5 mg/h patch, and 3 mg capsule) to ascertain practice-based medication familiarity. Information was entered in Microsoft Excel, coded, cleaned, and uploaded into Stata 10.1. where descriptive statistics and logistic regression analyses were performed.

## 3. Results

### 3.1. Characteristics of Respondents

A total of 862 responses (74%) were obtained from 1166 eligible community pharmacies and are summarized in Table 1. Respondents were balanced between male (50.3%) and female (49.7%). Just under half (43.5%) had terminal Bachelor of Science in Pharmacy (B.S.), while 56.5% had a Doctor of Pharmacy (Pharm.D.). One or more donepezil formulation(s) (donepezil 10 mg tablet, donepezil 23 mg tablet and/or donepezil 10 mg orally-disintegrating tablet) was in-stock in 88.6% of surveyed pharmacies. Rivastigmine formulations (rivastigmine 3 mg capsule and/or 9.5 mg/24 h patch) were in-stock in fewer pharmacies (28.6% and 47.1%, respectively). 

### 3.2. Pharmacists’ Knowledge Regarding Donepezil AEs

While over half of surveyed pharmacists (61.7%, *n* = 513) were able to name ≥1 AE, less than one third (31.4%, percent (*n* = 261) were able to name ≥2 AEs, and only 15.7 percent (*n* = 131) were able to name ≥3 AEs. (Table 2) Respondents’ ability to name individual, evidence-based AEs is shown in Table 3. Only four donepezil AEs were named in any significant numbers: nausea (36.1%); dizziness (25.1%); diarrhea (15.0%); and vomiting (13.9%). Other AEs were named by fewer than 7% of respondents (headache 3.8%, insomnia 6.4%, anorexia 6.6%, muscle cramps 1.4%, weight loss 1.6%, and fatigue 3.0%). Only 0.6% of all surveyed pharmacists named lower heart rate (*n* = 5), 0.2% lower blood pressure (*n* = 3), or 1.2% vivid dreams (*n* = 10). While the number of AEs named was low in all areas, logistic regression analyses identify that, compared to those in the ND/SD study area, respondents in the N.CA./S.OR. study area were more likely to name ≥2 AEs (OR = 1.70; 95% CI = 1.17–2.54) and ≥3 AEs (OR = 2.77; 95% CI = 1.62–4.74), and those in the West Virginia study area more likely to name ≥1 AEs (OR = 1.51; 95% CI = 1.07–2.14), ≥2 AEs (OR = 2.16; 95% CI = 1.57–2.98), and ≥3 AEs (OR = 2.86; 95% CI = 1.81–4.52). Overall, pharmacists with a Pharm.D. were more likely to name ≥1 AEs (OR = 1.65; 95% CI = 1.24–2.197), ≥2 AEs (OR = 1.86; 95% CI = 1.37–2.52), and ≥3 AEs (OR = 2.01; 95% CI = 1.34–3.01) compared to those with a B.S.

### 3.3. Pharmacists’ Knowledge Regarding Self-Care Recommendations

When providing a non-Rx sleep-aid recommendation, 12.4% of pharmacists (*n* = 107) provided inappropriate recommendations: valerian root and doxylamine, diphenhydramine, or another first-generation antihistamine. More than 20 percent of pharmacists were unable to provide any recommendation. Only 62.9% of pharmacists (*n* = 542) provided appropriate recommendations: melatonin (40.3%) or referral to physician (22.0%), along with sleep hygiene (0.6%). A small number of pharmacists (3.3%) refused to answer (Table 4). There was no difference among study areas with regard to provision of an inappropriate recommendation. Pharmacists with a Pharm.D. were less likely to make an inappropriate recommendation (OR = 0.43; 95% CI = 0.28–0.66), more likely to recommend melatonin (OR = 2.08; 95% CI = 1.56–2.77), and less likely to say “don’t know” (OR = 0.67; 95% CI = 0.48–0.93).

## 4. Discussion

Community pharmacists are highly accessible and, over the past few decades, their role has significantly expanded from traditional medication dispensing into provision of a variety of clinical services [11,12,17,21,22]. For community-dwelling persons with AD, there are potentially beneficial clinical services that community pharmacists/pharmacies can provide to ensure appropriate medication use and positive health outcomes [13,16,20]. Maintaining continuous pharmacological treatment of patients with Alzheimer’s disease can be difficult because of low persistence of treatment with AChEIs due to perceived medication inefficacy, unrealistic treatment expectations, and/or GI AEs [23,24,25,26]. Medication education and pharmacist-counseling have been associated with improved adherence and treatment persistence with AChEIs [27,28,29]. Inappropriate medications among community-dwelling persons with AD are relatively common including over-the-counter first-generation antihistamines with central anticholinergic AEs that can negatively impact cognition [30,31]. High-quality patient education on AChEIs and appropriate self-care recommendations for non-cognitive problems associated with dementia are a good starting point. 

Donepezil is a widely-prescribed cognitive enhancer because of its once daily dosing and FDA-approval for all AD stages [4]. Most surveyed pharmacies had donepezil in-stock. Donepezil, as well as other AChEIs, increase synaptic acetylcholine and postsynaptic cholinergic neurotransmission in the brain due to decreased breakdown of acetylcholine [4,5,6,7,32]. However, these medications also increase peripheral cholinergic activity that leads to GI AEs (i.e., nausea, vomiting, diarrhea, and/or anorexia) and bradycardia [4,5,6,7,33]. Additional AEs commonly reported with donepezil are dizziness, headache, weight loss, muscle cramps, and fatigue. These AEs are usually transient during dose initiation or increase [4,5,6,7]. A community pharmacist’s role is to provide medication education to patients and caregivers. All pharmacists practicing in the US should be able to provide effective counseling on medication AEs, more so for commonly stocked medications (i.e., donepezil). 

Despite donepezil being in-stock, knowledge regarding AEs among surveyed pharmacists was poor. While pharmacists with a Pharm.D. named more AEs than their B.S. peers, and pharmacists in the WV and N.CA./S.OR. study areas named more AEs than those in the ND/SD study area, neither degree nor area was associated with meaningfully improved knowledge regarding AEs. A patient or caregiver will typically see a community pharmacist after receiving a prescription for donepezil. Community pharmacists play a role in dispensing AChEIs but also providing medication-related information, especially important for those with filling a medication for the first time. Inadequate pharmacist knowledge regarding donepezil GI AEs can potentially result in patient distress or early discontinuation [27,29], or to lack of understanding on how to potentially decrease AEs (e.g., taking medication with food, dividing daily dose and taking separately). Patients or caregivers should be informed that these AEs are usually transient and improve with continuous medication use [4]. Lack of knowledge regarding donepezil AEs could reduce effectiveness of pharmacist counseling, negatively impacting adherence or treatment persistence [27,28,29].

Because of age-related physiologic changes, number of comorbidities, and/or co-administration of other medications, persons with AD might be at increased risk for donepezil interactions leading to potentially serious problems such as bradycardia, syncope and falls, bradyarrhythmia, and/or atrioventricular (AV) block [4]. Lower heart rate is rare; however, it can be a serious donepezil AE, especially for individuals with pre-existing bradycardia, AV heart block, or concomitant medications affecting heart rate (i.e., beta-blockers, non-dihydropyridine calcium channel blockers, or amiodarone) [4]. Only 5 (0.6%) surveyed pharmacists named this as an AE on which they would counsel a person with AD filling donepezil for the first time. 

An inappropriate recommendation of a first-generation antihistamine for management of insomnia in a person with AD treated with rivastigmine was made by 12.4% of pharmacists. There was no difference in rates of inappropriate recommendations among study areas, but pharmacists with a Pharm.D. were 57 percent less likely to make an inappropriate recommendation compared to those with a B.S. Given the central anticholinergic properties of this class of medications as well as AEs profile, this recommendation can negatively impact a patient [31]. Addition of diphenhydramine to a treatment regimen with rivastigmine (or AChEIs) is associated with reduced clinical effect of the cognitive enhancer due to central anticholinergic effect [6,7,30,34]. Diphenhydramine administration can also be associated with increased risk for cognitive AEs (i.e., memory and learning impairment), especially in older adults and those with pre-existing cognitive problems/deficit [31,35,36] and can actually worsen cognitive status and perceived efficacy of AChEIs, increasing risk for medication discontinuation. We also considered valerian root an inappropriate recommendation (*n* = 3) due to its pharmacologic properties that can increase risk for drug interactions, lower tolerability for older adults, and minimal clinical data on its use in dementia [36,37].

Melatonin was recommended by two out of five of pharmacists. Given the pathophysiology of AD and possible association of disrupted circadian rhythm [38], melatonin can be an appropriate recommendation that would not interact with disease state or medications [36,39,40,41]. However, efficacy of melatonin in AD has mixed results [37,42]. Over 20% of pharmacists were unable to provide any recommendation. Given community pharmacists’ role in self-care for community-dwelling individuals, this is substandard performance. While no harmful recommendation was made, neither was a helpful recommendation nor guidance offered. To provide an appropriate self-care recommendation for non-cognitive complications of AD, it is crucial for pharmacists to have knowledge regarding AD pathophysiology and treatment management so they can consider effectiveness, AEs, and interactions prior to providing a recommendation. Unfortunately, Rx and non-Rx medications with central anticholinergic effects are commonly used by community-dwelling older adults as well as persons with AD [30,31,43,44,45]. Pharmacists should play a role in decreasing anticholinergic drug prescribing for and utilization by vulnerable persons with AD. They should be proactive in increasing awareness regarding potential harm of these medications, recognizing and managing potentially inappropriate medications in persons with AD, and not contributing to this problem by making inappropriate recommendations. 

This study did not comprehensively assess AD knowledge but rather assessed two specific roles of community pharmacists: medication counseling and self-care recommendations, both part of care for community-dwelling persons with AD. The study was cross-sectional, thus was unable to comprehensively assess provision of complex AD care. Knowledge was not assessed for all cognitive enhancers. But as donepezil is the most widely available, knowledge is highly likely to better than for other AChEIs. 

## 5. Conclusions

Community pharmacists can play a beneficial role in AD-related pharmaceutical care, managing drug therapy through patient counseling and providing recommendations able to lead to optimal drug use and positive health outcomes. Knowledge of surveyed community pharmacists was grossly inadequate. Significant gaps in knowledge were identified regarding AChEIs. Given the large and still growing population with AD, the importance of medication counseling, and community pharmacists’ role in ensuring medication efficacy, tolerability, and safety for individuals with AD, knowledge improvement is needed regarding AChEIs including AEs, interactions, and self-care recommendations for insomnia in persons with AD. The findings seem to indicate a potential need for continuing education for community pharmacists in Alzheimer’s disease management. The authors are currently concluding a study on pharmacists’ decision-making, community information-seeking, and continuing education utilization related to AD.

## Figures and Tables

**Table 1:** Pharmacists Characteristics.

Characteristic	*n*	%
**Sex**		
**Male**	434	50.3%
**Female**	428	47.7%
**Pharmacy Terminal Degree**		
**Bachelor of Science in Pharmacy**	375	43.5%
**Doctor of Pharmacy**	487	56.5%
**Study Areas**		
**Northern California**	119	13.8%
**Southern Oregon**	58	6.7%
**North Dakota**	109	12.7%
**South Dakota**	156	18.1%
**West Virginia**	420	48.7%

**Table 2:** Pharmacists’ Knowledge of Donepezil Adverse Effects.

Knowledge of AEs	Mean	SD
Common AEs ^a^	1.17	1.27
Number of AEs Named	N	%
≥ 1 AEs	513	61.7%
≥ 2 AEs	261	31.4%
≥ 3 AEs	131	15.7%

Abbreviations: Adverse effects (AEs).^a^ Donepezil adverse effects with incidence rates of ≥5%: nausea, diarrhea, headache, vomiting, insomnia, anorexia, dizziness, muscle cramps, weight loss, fatigue [4].

**Table 3:** Pharmacists’ Knowledge of Individual Donepezil Adverse Effects.

**Common Adverse Effects ^a^**	***n***	**%**
Nausea	311	36.1%
Diarrhea	129	15.0%
Headache	33	3.8%
Vomiting	120	13.9%
Insomnia	55	6.4%
Anorexia	57	6.6%
Dizziness	216	25.1%
Muscle Cramps	12	1.4%
Weight Loss	14	1.6%
Fatigue	26	3.0%
**Other Adverse Effects ^b^**	***n***	**%**
Bradycardia ^c^	5	0.6%
Vivid Dreams	10	1.2%

^a^ Adverse effects with reported incidence rates of ≥5%: nausea, diarrhea, headache, vomiting, insomnia, anorexia, dizziness, muscle cramps, weight loss, fatigue [4]. These adverse effects are also listed in order from highest to lowest reported incidence rate; ^b^ Adverse effects with reported incidence rate <3% [4]; ^c^ Rare but severe adverse effect [4].

**Table 4:** Pharmacists’ Non-Prescription Sleep-Aid Recommendations.

Recommendation	*n*	%
**APPROPRIATE**	542	62.9%
**Melatonin**	347	40.3%
**Refer to Physician**	190	22.0%
**Sleep Hygiene**	5	0.6%
**INAPPROPRIATE**	107	12.4%
**Anticholinergic Agent ^a^**	104	12.1%
**Valerian Root**	3	0.4%
**DON’T KNOW**	185	21.5%
**REFUSE**	28	3.3%

^a^ Anticholinergic agent: any over-the-counter product containing diphenhydramine, doxylamine, or first generation antihistamine

## Supplementary Materials

The following are available online at http://www.mdpi.com/2226-4787/5/3/42/s1, (1) Research Dataset.
